# Temperate conditions restrict Japanese encephalitis virus infection to the mid-gut and prevents systemic dissemination in *Culex pipiens* mosquitoes

**DOI:** 10.1038/s41598-021-85411-2

**Published:** 2021-03-17

**Authors:** Arran J. Folly, Daniel Dorey-Robinson, Luis M. Hernández-Triana, Stuart Ackroyd, Beatriz Vidana, Fabian Z. X. Lean, Daniel Hicks, Alejandro Nuñez, Nicholas Johnson

**Affiliations:** 1 Arbovirus Research Team, Virology Department, Animal and Plant Health Agency, Woodham Lane, Addlestone, Surrey KT15 3NB UK; 2 Pathology Department, Animal and Plant Health Agency, Addlestone, Surrey KT15 3NB UK; 3 Faculty of Health and Medicine, University of Surrey, Guildford, Surrey GU2 7XH UK; 4 Present Address: Pirbright Institute, Ash Road, Woking, Surrey GU24 ONF UK; 5 Present Address: Bristol Veterinary School, University of Bristol, Langford House, Langford, Bristol BS40 5DU UK

**Keywords:** Viral vectors, Viral transmission

## Abstract

Japanese encephalitis virus (JEV), a mosquito-borne flavivirus, is the main cause of viral encephalitis in Asia. However, with changing climate JEV has the potential to emerge in novel temperate regions. Here, we have assessed the vector competence of the temperate mosquito *Culex pipiens f. pipiens* to vector JEV genotype III at temperatures representative of those experienced, or predicted in the future during the summer months, in the United Kingdom. Our results show that *Cx. pipiens* is susceptible to JEV infection at both temperatures. In addition, at 25 °C, JEV disseminated from the midgut and was recovered in saliva samples, indicating the potential for transmission. At a lower temperature, 20 °C, following an incubation period of fourteen days, there were reduced levels of JEV dissemination and virus was not detected in saliva samples. The virus present in the bodies of these mosquitoes was restricted to the posterior midgut as determined by microscopy and viable virus was successfully recovered. Apart from the influence on virus dissemination, mosquito mortality was significantly increased at the higher temperature. Overall, our results suggest that temperature is a critical factor for JEV vector competence and infected-mosquito survival. This may in turn influence the vectorial capacity of *Cx. pipiens* to vector JEV genotype III in temperate areas.

## Introduction

Flaviviruses have a significant global distribution, many of which are transmitted by mosquitoes and other invertebrates such as ticks and sandflies^1^. There have been repeated incidences of flavivirus emergence in novel environments^2–4^ and this is likely a consequence of their ability to co-opt multiple vectors^5^. Japanese encephalitis virus (JEV) (genus *Flavivirus*, family *Flaviviridae*) consists of an enveloped positive-sense single stranded RNA genome, approximately 11 kilobases in length, and is the main causative agent of human viral encephalitis in Far East and South East Asia^6^. Five genotypes are recognised based on genomic phylogeny and each has a distinct distribution in Asia that may be related to climatic conditions^7^. Typically JEV is maintained in a natural enzootic cycle between mosquito vectors and avian reservoirs^8,9^. However, as some vectors of JEV are not host specific they can act as bridge vectors^10,11^, resulting in transmission to, and infrequent clinical disease in both livestock, where pigs act as amplifying hosts, and humans, who are considered dead-end hosts. In addition, given that current climatic models predict global temperature rises and shifts in climatic zones^12^, combined with the increased global movement of people and livestock more generally, it seems likely that JEV could emerge in novel areas in the future^13,14^. Consequently, understanding the capacity of mosquitoes within non-endemic areas to vector JEV is critical in designing effective disease intervention strategies.

The principal mosquito species associated with JEV transmission is *Culex tritaeniorhynchus* although a wide range of other species, particularly within the genus *Culex* can act as a vector where they are locally abundant^15^. In Europe, *Cx. tritaeniorhynchus* has only been reported in Greec^16^. However, *Cx. pipiens* is reported across the continent and is associated with transmission of other flaviviruses such as West Nile virus and Usutu virus^17^. Within the UK there have been no autochthonous mosquito transmitted viruses^18^, which is likely a result of the depauperate indigenous vector diversity, temperate climate and the geographic barrier that the English Channel provides against the emergence of diseases or their vectors^19^. Nevertheless, incidences of arbovirus emergence have been recorded in the UK, including bluetongue virus^20^, Schmallenberg virus^21^ and more recently Usutu virus was detected in wild birds^22^. Furthermore, in Europe the spread of West Nile virus has had a significant impact on wildlife, livestock and human populations^23^. Consequently, there is a continuing risk to public and animal health should a virus emerge in the UK that can be transmitted by the indigenous mosquito population. Interestingly, a number of UK mosquito species have been identified as being competent vectors for flaviviruses^24,25^. One species, *Aedes detritus*, originating in the UK has been shown to be competent to transmit JEV genotype V, although this mosquito species is restricted to coastal locations^26^. A more commonly distributed species and a more likely vector is *Culex pipiens*, a mosquito species that has been associated with transmission of a range of arboviruses^27,28^. In addition recent investigations have confirmed that *Cx. pipiens* from France^29^, China^30^ and the UK^31^ are competent to transmit JEV.

A key factor in mosquito mediated transmission of pathogens is environmental temperature. Mosquitoes are ectothermic and consequently many aspects of their biology are influenced by temperature. This extends to virus infection and extensive studies have demonstrated the role of temperature on flavivirus transmission by *Culex* spp.^32,33^. In general, increasing temperatures enhance vector competence for flaviviruses, peaking between 23 and 29 °C^34^. However, elevated temperatures can have detrimental effects on the survival of some mosquito populations. Consequently, it has been suggested that higher temperatures can reduce overall vector capacity^35^. In order to demonstrate the vector competence of UK *Cx. pipiens* for JEV genotype III and to investigate the impact of temperature on virus infection, we provided an infectious bloodmeal to a laboratory maintained colony of *Cx. pipiens.* Blood fed individuals were separated and monitored for fourteen-days at both 20 °C and 25 °C, as these temperatures are representative of temperate summer conditions. Given that *Culex* species are effective vectors of arboviruses globally and that increased temperatures enhance vector competence, we predict that UK *Cx. pipiens* will be competent to transmit JEV genotype III at these temperatures.

## Methods

### Mosquito provenance

A colonised line of *Cx. pipiens* f. *pipiens* Caldbeck UK (hereafter referred to as *Cx. pipiens*) (f > 100 generations) (donated by the Pirbright Institute, UK, and characterized by^36^) were kept in a dedicated insectary following the protocol of^25^. In essence, mosquitoes were provided a photo-period of 12:12 (light:dark), and room conditions were maintained at 24 ± 2.5 °C and 50% humidity, as these conditions resulted in successful mosquito husbandry for this colony. Adult mosquitoes were housed in a bugdorm insect cage (245 × 245 × 245 mm) (maximum 200 mosquitoes per cage) and provided cotton wool saturated in 10% (w/w) sucrose solution which they could feed ad libitum. In addition, adult mosquitoes were blood-fed twice a week with defibrinated horse blood (TCS Biosciences, UK) using a Hemotek system (Hemotek, Blackburn, UK) with a parafilm membrane, to encourage egg laying. Black plastic cups filled with distilled water were provided to each cage for oviposition and eggs were collected and transferred to rearing trays twice a week. Larval rearing trays comprised a plastic tub (280 × 280 × 110 mm) filled two thirds with distilled water and covered with a muslin cloth using a rubber band. Larval rearing tray water was changed weekly, and larvae were fed using commercially available Guinea pig food twice per week, or once the food had been consumed, whichever came first. Newly eclosed adults were transferred to the cages (described above) daily using a battery powered aspirator (Katcha, UK). When colony progeny had reached sufficient levels for the experiment (n = 285 females that had all eclosed within the last seven days), a bugdorm cage (described above) was used to transfer all the experimental mosquitoes (hereafter referred to as mosquitoes) to a dedicated Bio-safety level 3 (BSL-3) laboratory.

### Evaluation of vector competence of *Cx. pipiens* for Japanese encephalitis virus genotype III

All subsequent procedures, unless explicitly stated, were undertaken in a BSL-3 laboratory at the Animal and Plant Health Agency, Weybridge, UK. Vector competence studies allow inferences on whether a particular vector can become infected by a pathogen, resulting in a disease phenotype. In addition, vector competence studies facilitate the detection of disseminated infections, where a disease has moved from the initial infection site and in turn whether the vector can then transmit a pathogen to a naïve host. Within this study system, should an infection disseminate to the salivary glands and be detected in the saliva then the mosquito vector is presumed competent to transmit an infection during blood feeding. A strain of JEV genotype III (SA14, isolated from *Culex pipiens* larvae, China 1954, donated by Pr. Dr. Jonas Schmidt-Chanasi, Bernhard Nocht Institute for Tropical Medicine, Hamburg, Germany) was used for oral inoculations and was selected as it is a well characterised strain^37^, which is representative of the prevalent JEV genotype III and is associated with temperate climates^7^. Consequently, this strain was an ideal choice for assessing the vector competence of a temperate mosquito. Virus was propagated in Vero E6 cells in 25 ml of a culture medium consisting of Eagles minimal essential medium (E-MEM-Sigma Aldrich, UK), with 10% foetal bovine serum (FBS) and Penicillin–Streptomycin-Nystatin solution (1% Thermo Fisher Scientific), at 37 °C and 5% CO^2^ for three days in T75 flasks. Viral titre was ascertained by plaque assay, following the protocols of Hernández-Triana et al.^25^. The concentration of the propagated JEV SA-14 strain was calculated to be 5.5 × 10^6^ plaque forming units (PFU/ml). Stocks were stored at − 80 °C until required.

Prior to oral inoculation, sucrose feeders were removed from cages and mosquitoes were maintained on water saturated cotton wool for 16 h and then starved of water for three hours to encourage feeding during virus challenge. To inoculate, mosquitoes were offered blood meals overnight in a darkened bugdorm cage (by surrounding the cage with tin foil to block out any light) using a Hemotek, as described above. The blood inoculum was created using defibrinated horse blood (TCS Biosciences, UK) and contained 1.8 × 10^6^ PFU/ml of JEV SA-14 (as calculated above), which falls within the natural range of livestock viremia ^9^ (in a 2:1 blood: virus ratio). The blood inoculum also contained 1 µM dATP (Thermo Fisher Scientific, UK) to stimulate mosquito feeding. Blood meal samples were taken before and after feeding for confirmation of virus titre and stored at -80 °C.

Post feeding, mosquitoes were anesthetised using FlyNap (Carolina Biological Supply, Burlington, US) and engorged mosquitoes were sorted into groups of ten (n = 10 groups per temperature variable) and placed into 73 × 118 mm micro-habitat pots with a mesh vent. These pots were kept in one of two dedicated incubators, either set to 20 °C (n = 10 pots) or 25 °C (n = 10 pots) for the duration of the experiment. Mosquitoes were monitored daily and fed ad libitum with water and Manuka honey using saturated FTA cards (Whatman, UK), which were replaced when required. Used FTA cards were stored for subsequent viral RNA analysis at − 80 °C. Sampling of mosquitoes occurred at the following time points, 0 days post oral inoculation (d.p.i), 7 d.p.i and 14 d.p.i. This range provided a realistic timeframe for virus to infect and replicate inside a naïve mosquito if it was susceptible to infection^38^. For sampling, mosquitoes were anesthetised using FlyNap. Once incapacitated, legs and wings were removed from each mosquito, using a sterile pair of forceps, to screen for evidence of virus dissemination. These were placed into a 2 ml collection tube containing 5 mm ceramic beads and 300 µl experimental E-MEM (described above). In addition, saliva was collected to assess the transmission potential of *Cx. pipiens*. This was achieved by placing the proboscis of the mosquito into a trimmed 200 µl pipette tip containing 30 µl of experimental E-MEM (described above). Following this, 10 µl of pilocarpine mix (Pilocarpine hydrochlorides (Thermo Fisher Scientific, UK), 0.1% Tween 80 in PBS (Sigma-Aldrich, UK)) was applied to the abdomen of the mosquito to encourage salivation^39^. After thirty minutes of exposure, the media containing the expectorated saliva was combined with 270 µl of experimental media (described above). Finally, the mosquito body was collected and stored in the same manner as the legs and wings (described above). To homogenise, each sample was subjected to a one-minute 4500 rmp cycle on a QiaGen Tissue lyser. All homogenised samples were then centrifuged at 3000×*g* for 10 mins.

### Viral RNA extraction and reverse transcription-polymerase chain reaction (RT-PCR)

Viral RNA was independently extracted from each of the collected samples using the TRIzol or TRIzol LS kit (Thermo Fisher Scientific) and the resulting RNA was eluted in 20 µl of RNAse free water before being stored at − 80 °C until required. Each sample was initially screened for JEV viral RNA by Reverse Transcription-Polymerase Chain Reaction (RT-PCR) on an MX Pro 3000 RT-PCR system (Agilent Technologies, US) using primers described previously^40^ and the IScript TaqMan master mix (Thermo Fisher Scientific) with 1 µl template. Cycling conditions comprised a RT step at 50 °C for 10 min, a RT inactivation at 95 °C for 5 min and 45 cycles of 95 °C (15 s) and 55 °C (30 s). Amplification curves were visualised in MX3000p v4. software (Agilent Technologies, US).

### Virus isolation and titration

Any positive samples, which may indicate vector competence, were then subjected to virus isolation. Virus isolation was achieved by mixing 20 μl of sample (body or leg clarified homogenate or saliva sample) with 80 μl Vero E6 cell suspension and added to a 96 well plate in duplicate. Plates were incubated at 37 °C supplemented with 5% CO_2_ for 3 days and monitored for cytopathic effect. This was confirmed by fixing the monolayer in 80% acetone for 20 min and then staining with crystal violet (Sigma-Aldrich, UK) solution diluted 1 in 100 in phosphate buffered saline.

Virus titration of pre and post bloodmeal samples were undertaken in 12-well plates seeded with confluent Vero E6 cells as follows. A 50 µl aliquot of virus dilution was absorbed for 1 h at 37 °C in each well. An avicel overlay (0.5 ml 0.6% avicel in MEM, Sigma-Aldrich, UK) was applied to each well and incubated for seven days in a dedicated incubator set to at 37 °C with 5% CO_2_. Cells were fixed in 10% formalin for one hour and then stained to aid visualisation with 0.2% Crystal violet^25^.

### Virus genomic sequencing

A Nextera XT DNA library preparation kit (2 × 150-bp reads; Illumina, San Diego, US) was used for library preparation from extracted RNA. Sequencing was carried out on an Illumina MiSeq sequencer. The reads were aligned, and genome completeness assessed against the JEV SA14 genome (GenBank accession number KU323483, length = 10,977 bp, GC content = 51.43%). Both Burrows-Wheeler Aligner (BWA) v0.7.13^41^ and SAMtools v1.9^42^ were used to iteratively construct a consensus sequence, which was visually inspected using Tablet^43^.

### Immunohistochemical detection of JEV III in *C. pipiens*

The detection of JEV antigens in infected mosquitoes was determined by immunohistochemistry (IHC) in histological sections. Fourteen infected blood-fed and six non-infected control female mosquitoes, from our 20 °C group were fixed in 10% neutral buffered formalin for 48 h. After fixation, the wings and legs were removed and the body was positioned into a sagittal plane prior to routine processing and paraffin embedding. Serial 3-μm-thick sections of the formalin-fixed paraffin-embedded (FFPE) mosquitoes were cut and placed on to silane-coated slides (3-trietoxysilyl-propylamine). Proteinase enzyme buffer (DAKO, Ely, Cambridgeshire, UK) was then applied for 15 min at 20 °C to retrieve antigen. Slides were washed in distilled water and assembled into cover plates for immunolabeling. Following this, a mouse monoclonal anti-Flavivirus E-glycoprotein antibody (ab155882, Abcam, Cambridge, UK) diluted in Tris-buffered saline with 0.05% Tween 20 (TBST, VWR, Leicestershire, UK) at 1:50 dilution, with 0.05% Tween 20 (TBST, VWR, Leicestershire, UK) was applied at 4 °C for 18–20 h (overnight). This antibody was used as the primary indicator to detect JEV in our sections. A serial section was stained with a concentration matched, normal mouse immunoglobulin G class 2a (Abcam, Cambridge, UK), as an isotype control to assess non-specific immunolabeling.

DAKO mouse EnVision + System, HRP Peroxidase (DAKO, Ely, Cambridgeshire, UK) was used as a secondary antibody and amplifier, this was incubated for 30 min at 20 °C and combined with swine and goat immune serum (Vector Laboratories, Peterborough, UK). Antibody binding was visualized using the mouse Envision-kit from DAKO (DAKO, Ely, Cambridgeshire, UK) and the chromogen 3,3′-diaminobenzidine (DAB) + substrate-chromogen with 10 min of incubation. Finally, sections were counterstained with Mayer’s haematoxylin (HE) and mounted in Distyrene Plasticiser Xylene (DPX) mounting medium (TCS Bioscience, Buckingham, UK) for light microscopy. A FFPE from a known West Nile virus infected mouse brain and JEV infected Vero-infected cells were used as positive controls for flavivirus immunostaining.

### Statistical analysis

All graphical outputs were undertaken in R programming language (http://www.R-project.org). A two-way ANOVA and a Tukeys post-hoc test was used to compare cycle threshold (*ct*) values for RT-PCR analysed mosquito bodies, leg/wings and saliva samples from the 20 °C and 25 °C treatments and a chi-squared test was used to compare overall mortality between treatment groups.

## Results

### JEV vector competence

**Figure 1:**
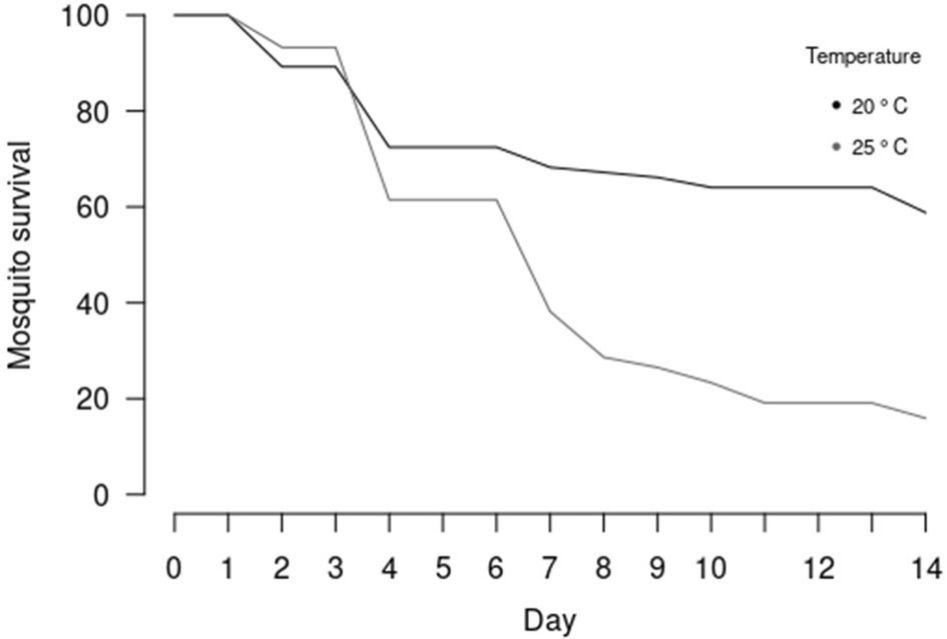
Survival of *Cx. pipiens* following a blood-meal containing JEV over the fourteen-day experimental timeframe. Mortality was two-fold higher in the 25 °C cohort when compared with the 20 °C cohort (χ^2^ = 52.68, *P* < 0.001).

To assess vector competence of *Cx. pipiens* mosquitoes indigenous to the UK, 285 females were fed on a bloodmeal containing JEV (1.8 × 10^6^ pfu/ml). A total of 255 females successfully fed (255/285, 89%) and these were divided into groups (n = 10) that were held for 14 days at two temperatures. A total of 120 females were held at 25 °C, which is higher than the historical average maximum temperature for the south of England (see Supplementary Data Table S1) and 135 females were held at 20 °C, which is below the historical maximum and more commonly experienced in the UK. In the 20 °C group, 42% of mosquitoes died during the experiment leaving 70 mosquitoes for downstream analysis. In contrast, mortality in the 25 °C group reached 85% during the 14-day experiment, leaving only 20 mosquitoes for downstream analysis. Consequently, overall mortality in the 25 °C group was significantly higher when compared to the 20 °C group (χ^2^ = 52.68, *P* < 0.001) (Fig. 1).

Virus infection, dissemination and transmission, in this case assessed by the presence of virus (genome) in saliva, was initially determined for both groups using RT-PCR. A total of 56 mosquitoes had total RNA extracted in the 20 °C group and due to a high mortality rate, 20 mosquitoes were analysed from the 25 °C group. In the 20 °C group JEV RNA was detected from 39 mosquito body samples. In addition, JEV RNA was also recovered from the legs and wing samples from five mosquitoes. No JEV RNA was recovered from any of the saliva samples in the 20 °C group. In contrast, JEV RNA was recovered from 18 out of 20 mosquito bodies in the 25 °C group and from 14 of these we also recovered JEV RNA in both the leg and wing samples and the expectorated saliva samples (Table 1). The presence of JEV in these samples was confirmed by virus isolation in Vero cells (Supplementary Data Fig. S1). This confirmed the presence of replicating virus in the bodies of mosquitoes held at 20 °C and for dissemination to the legs and wings in a small number of mosquitoes, corroborating the observations based using RT-PCR (Table 1).

**Table 1:** The proportion of *C. pipiens pipiens* samples for which JEV RNA was recovered in both 20 °C and 25 °C treatments 14 days post inoculation.

Sample	20 °C treatment	25 °C treatment
Body (infection)	39/56 (69.6%)	18/20 (90%)
Legs and wings (dissemination)	5/40 (12.5%)	14/20 (70%)
Expectorated saliva (transmission)	0/40 (0%)	14/20 (70%)

From the 70 mosquitoes sampled from the 20 °C treatment, 14 were sent for IHC analysis and during experimental manipulation 16 mosquitoes were damaged so that leg and wings, and saliva samples could not be obtained.

**Figure 2:**
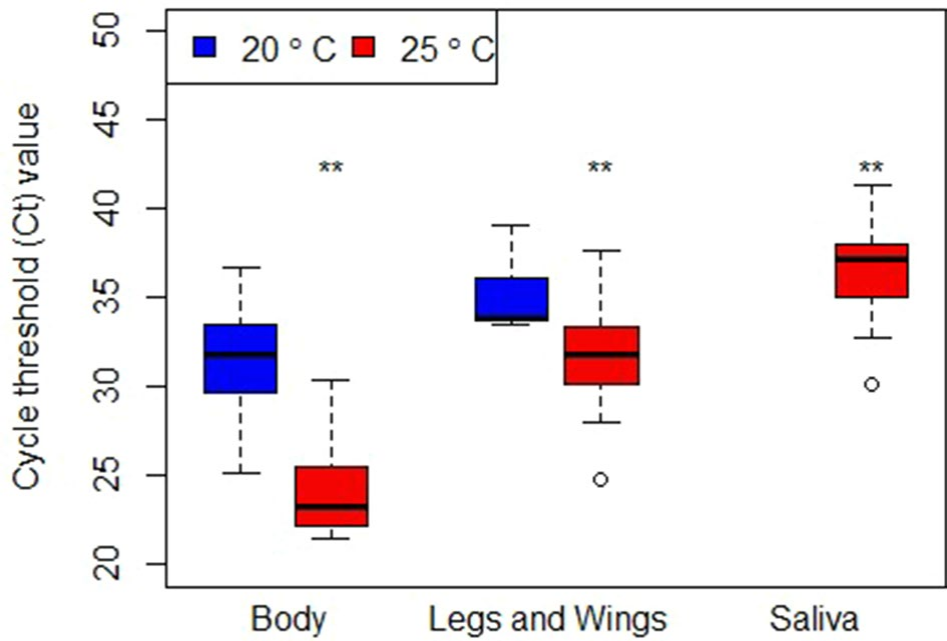
Cycle threshold (*ct*) values for recovered JEV RNA from *Cx. pipiens* body (n = 39 and 18), leg/wing (5 and 14) and saliva samples (0 and 14) in both the 20 °C and 25 °C treatments respectively, significant differences between temperature treatments have been marked with a double asterisk. Viral RNA was only recovered from the saliva of mosquitoes in the 25 °C treatment, suggesting that these mosquitoes were competent to transmit JEV.

In order to compare the relative amounts of virus genome in different samples the threshold value from each amplification cycle threshold (*ct*) were compared. Values recorded for JEV RNA from the 25 °C group were significantly lower than those recovered from the 20 °C group across body, leg/wings, and saliva samples (two-way ANOVA, *F* = 10.02, *P* = 0.002) (Fig. 2). These results indicate that JEV RNA was present at higher levels in the 25 °C group, which corroborates the impact of temperature on viral levels identified in other vector competence studies using *Cx. pipiens*^36^. In addition *ct* values were significantly different between sample types (two-way ANOVA, Tukeys pairwise comparison leg/wings and body *P* < 0.001, saliva and body *P* < 0.001, saliva and leg/wings *P* < 0.001). In essence, lower *ct* values and therefore higher genome levels were found in the body, but *ct* value increased and genome levels decreased in both the leg/wing and saliva samples. Finally, a significant interaction was identified between temperature and sample type (two-way ANOVA, *F* = 5.05, *P* = 0.02) whereby *ct* and sample type is dependent on temperature. While we recorded a significantly higher mortality rate in the 25 °C group, we also recorded a higher proportion of infected mosquitoes, which critically contained JEV RNA and viable virus in their saliva samples, indicating that *Cx. pipiens* may be competent to vector JEV under these conditions.

Two saliva samples with the highest quantitative RT-PCR value (assessed using *ct* values, saliva sample 1 *ct* = 28.55, and saliva sample 2 *ct* = 30.84) were selected for whole genome sequencing to evaluate for genomic changes during infection within the vector. From both samples consensus sequences for complete JEV genomes were generated (10,977 bp) that had well supported read-coverage depths (Supplementary Data Fig. S2). For saliva sample one the resulting contig (10,977 bp) consisted of 209,905 reads, with an average read depth of 2224 and a GC content of 51.36%, whereas the resulting contig for saliva sample five (10,977 bp) consisted of 25,320 reads, with an average read depth of 231 and a GC content of 51.37%. No variation in consensus sequences was identified between the stock virus used to infect the mosquitoes, virus obtained from the blood meal post-feeding and the virus recovered from both saliva samples using NGS.

### Immunohistochemical detection of JEV in infected *Culex pipiens*

**Figure 3:**
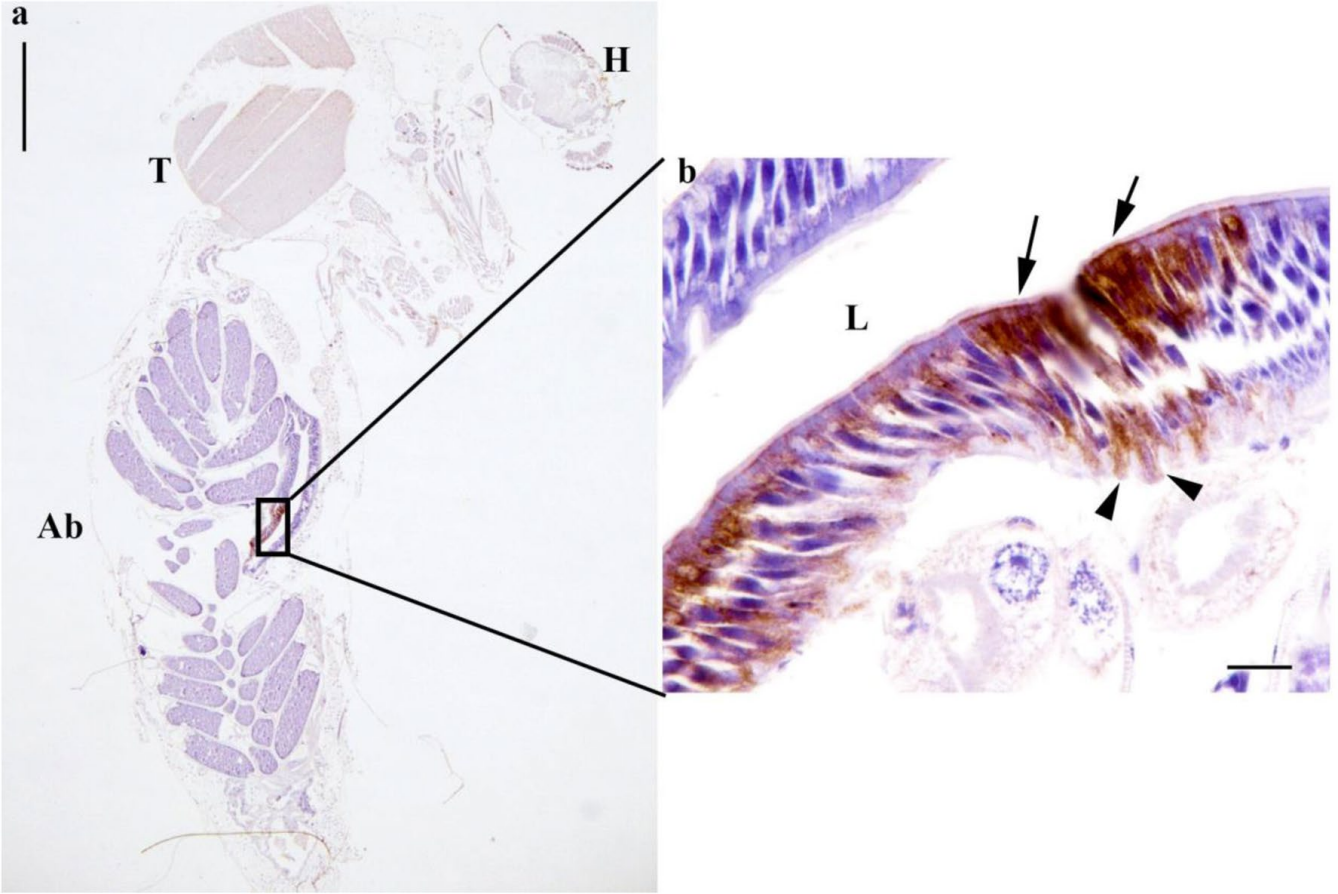
JEV infection of posterior midgut epithelial cells in the *C. pipiens*. Microscopic features of a blood-fed female mosquitoes. Head (H), thorax (T), abdomen (Ab). Region of interest outlined (rectangle). Scale bar: 500 µm (**a**). Brown intracytoplasmic immunolabelling in the posterior midgut of apical ciliated cells (arrow) and basal epithelial cells (arrowhead) Lumen of the midgut (L). Scale bar: 20 µm (**b**).

**Figure 4:**
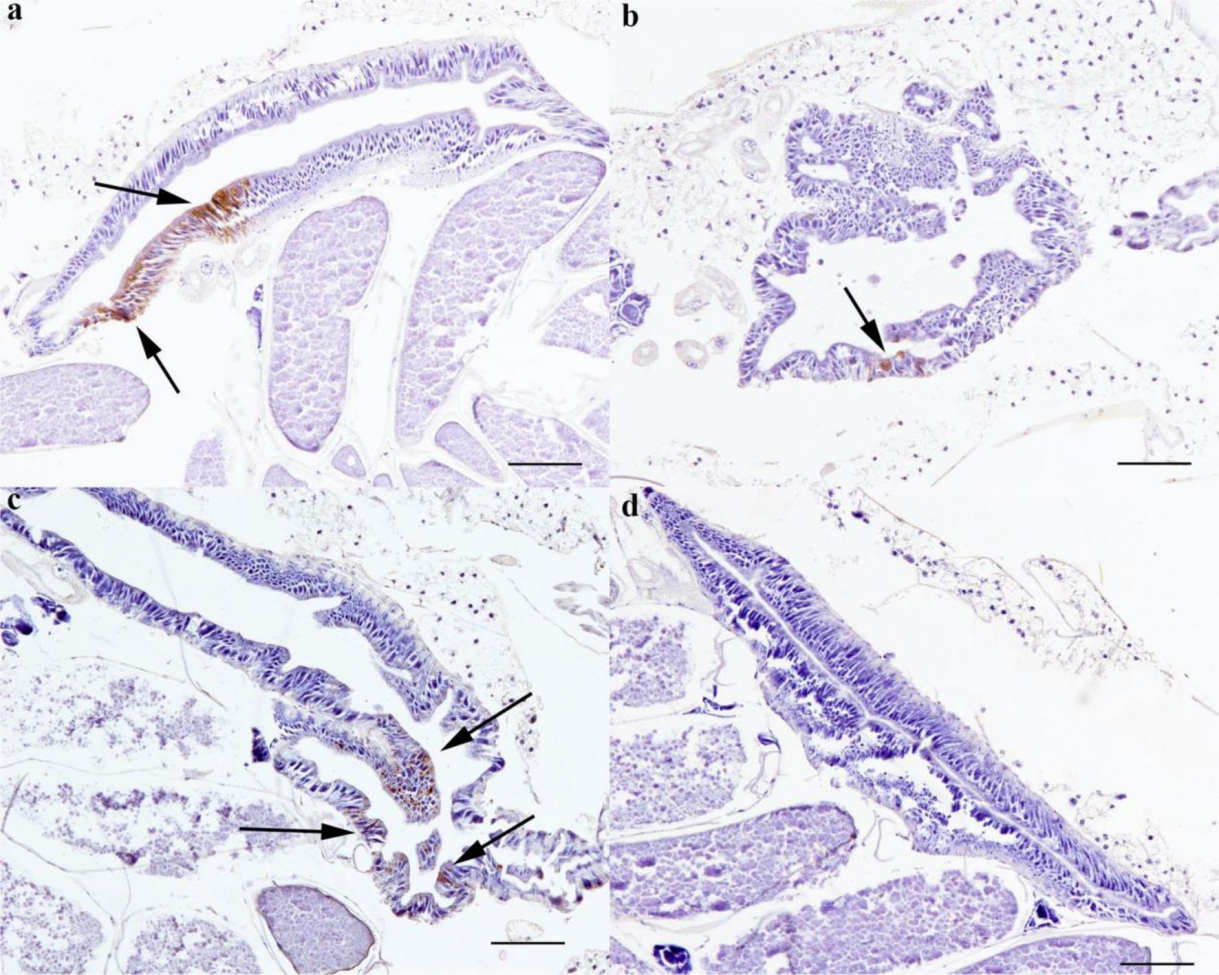
Variability of midgut infection by JEV in *C. pipiens.* Positive immunolabeling observed in posterior midgut of infected blood-fed mosquitoes (**a**–**c**) and unsuccessful infection (**d**). There is variability in the number of positive immunostained cells. Some mosquitoes showed high numbers of immunolabeled cells (**a**), singly immunostained epithelial cells (**b**, **c**) and no positive labelling (**d**). Scale bar: 100 µm.

Although viable virus was recovered from the bodies of our experimental mosquitoes, limited dissemination and absence of virus in saliva samples within the 20 °C group suggested an infection barrier within the mosquitoes at this temperature. To determine the cellular distribution of JEV infection in mosquitoes held at 20 °C, IHC was performed on sections of fourteen FFPE whole mosquitoes to detect envelope antigen. All segments of the mosquito, head, thorax and body, were examined by light microscope (Fig. 3a). One of the infected specimens was damaged during histological processing and therefore was not suitable for evaluation. Positive JEV immunolabeled cells were observed in the posterior midgut of eight infected females from the 20 °C treatment (Fig. 3b). The immunolabelling was present in clusters of epithelial cells, predominantly ciliated pseudostratified intestinal cells, located in the posterior midgut region, as characterised by dark brown pigment deposition within the cytoplasm. The staining was also present in small population of basal cells (Fig. 4). The levels of midgut epithelial cell infection is highly variable with labelling ranging from a continuous row of cells (Fig. 4a) to single cells (Fig. 4b,c) and no immunolabelling (Fig. 4d). No positive immunolabelling was observed in any other organs, including the salivary gland, corroborating the detection of virus by other methods (Table 1). Isotype control immunolabelled sections did not show any non-specific staining on infected mosquitoes (Supplementary Data Fig. S3).

## Discussion

Our results show that the temperate UK mosquito *Cx. pipiens* is susceptible to infection by JEV genotype III. In addition, our results demonstrate that at 25 °C after fourteen days the virus is able to disseminate throughout the mosquito with viable virus being recovered from mosquito saliva, suggesting that *Cx. pipiens* may be a competent vector for JEV genotype III, under this experimental paradigm. This corroborates experimental findings from other *Cx. pipiens* populations from the UK, Europe and Asia^29–31^. Given that we recorded such a marked increase in infection and dissemination prevalence at 25 °C it is likely that the limiting temperature at which *Cx. pipiens* becomes unable to transmit JEV genotype III, within fourteen days, falls between our two treatments^44^. However, our results also show that higher temperatures were associated with increased mortality in infected mosquitoes, when compared to mosquitoes in the 20 °C treatment. Together these results suggest that while *Cx. pipiens* may be a competent vector for JEV, elevated temperatures required for disseminated infection, potentially limiting the ability of JEV to emerge in temperate areas as greater numbers of vectors may succumb to the detrimental effects of infection prior to successful virus transmission events.

For both the 20 °C and 25 °C treatments JEV was recovered from mosquito bodies fourteen days post oral inoculation. The lifespan of *Cx. pipiens* is temperature dependent and for both treatments fourteen days is representative of approximately 50% of the average lifespan of females^38^. To facilitate disease transmission a vector must be susceptible to infection and infection must disseminate and have time to be transmitted to a new host. Consequently, fourteen days would still allow sufficient time for a mosquito to blood feed and act as a vector post experiment and therefore presents a meaningful timeframe from which competence can be assessed in this system. However, within the 20 °C group dissemination was only recorded in 12.5% of mosquitoes and no infectious virus or viral RNA was detected in any of the saliva samples. Combined, these results show that mosquitoes from our 20 °C treatment were not competent to transmit JEV after fourteen days. In contrast, JEV RNA was recovered from both extremities and saliva from 70% of mosquitoes from the 25 °C treatment. In addition, JEV consensus sequences were successfully constructed from NGS reads from saliva samples. While no sequence variation at the consensus level from a single passage was identified, low level mutation has been identified within the genome of the closely related flavivirus, WNV in *Cx. pipiens* following repeated infection^45^ in which multiple chain of transmission is more resemblance of natural transmission. Consequently, investigating how repeated transmission in vectors may impact viral evolution and epidemiology with JEV warrants further investigation. Interestingly, Chapman et al.^31^ reported the recovery of JEV RNA from 72% of infected *C. pipiens* saliva samples at 18 °C, whereas we did not recover any JEV RNA in saliva at 20 °C. It is important to note that Chapman et al. collected saliva at day 21 and the infection paradigm used JEV strain CNS138-11, which is representative of JEV genotype II^37^. Combined with our results and given that different genotypes of JEV are differentially circulating in South East Asia and Australia it is likely that vectors have variable competence for virus transmission and that this is likely influenced by viral genotype, temperature and extrinsic incubation period.

JEV is endemic throughout South East Asia with repeated incursions into north east continental Australia^8,46^. These areas are typified by a tropical, humid climate with consistently high temperatures unlike temperate regions. However, climatic changes in temperate zones have resulted in longer warmer summers^12^. Therefore, the likelihood of extended time periods at temperatures that fall between our treatments occurring during the summer months in temperate regions is becoming increasingly feasible. In addition, these conditions enable mosquitoes, and arthropods in general, to remain active for a longer period of the year, increasing the likelihood of transmission events and impacting disease epidemiology more generally^22^. We would note that our experimental temperature conditions maintained constant heat and humidity with a 24-h day night photoperiod (12:12), which is typical of vector competence studies^25,28,47^. However, these conditions are not representative of temperate regions as we have not accounted for diurnal shifts in temperature and humidity. Given that our results suggest that temperature appears to be critical for increasing both vector competence and vector mortality in this system, incorporating diurnal shifts more representative of current and projected conditions may also influence vector competence.

Virus antigen labelling shows that at the 20 °C treatment, JEV was only able to infect posterior midgut epithelial cells such as intestinal stem cells, which corroborates our detection of virus in the mosquito body. It also supports a previous study showing that midgut epithelial cells are among the first cells to support viral replication^48^. In addition, in the analysed mosquitoes no viral antigen was observed in any other organ by IHC, indicating that at this temperature, fourteen days post bloodmeal, the virus was not able to overcome the midgut barrier to spread systemically and to infect secondary organs such as the salivary glands. The virus present in the midgut appeared viable by the recovery of live virus in vitro from homogenised mosquito bodies. It is therefore unclear from these observations whether the restriction to the midgut was a result of active anti-viral control by the mosquitoes or the lower temperature alone restricting virus replication^49^. It is possible that an increase in temperature, or an increase in the duration of the experiment^31^ could trigger further virus replication and escape from the midgut. These results highlight the important role of midgut cells in mosquito-resistance and control of arboviruses, especially JEV infection in this model, which has been shown for other pathogens, including other *Flaviviridae*^48,50^.

Population results for both treatments show that mortality was two-fold higher in the 25 °C group compared to the 20 °C group and this could have a fundamental impact on the vectorial capacity of a mosquito species if this is due to increased virulence of the virus at higher temperatures^35^. To transmit from a vector to a new host a virus typically has to infect, replicate, and disseminate inside a vector. Incidentally, viral infection can have a negative impact on vector fitness, especially if the virus is particularly virulent^51^. The colonised line of *Cx. pipiens* used in this study is maintained under similar conditions to our experimental treatments and these do not have an adverse effect on longevity. However, in both groups mortality was higher than what would be expected for natural background levels (Animal and Plant Health Agency unpublished data). Consequently, active JEV infection, which we have identified here, may be having a detrimental impact on *Cx. pipiens* longevity and fitness at higher temperatures, as seen by comparing mortality data from the 20 °C treatment with the 25 °C treatment. Given that we recovered a higher prevalence of infection, and a higher rate of dissemination in the 25 °C group, it is likely that virus titre was higher in these mosquitoes and therefore the impact of infection is also likely to be higher. Indeed, the impact of higher viral titres on vector mortality has previously been identified in temperate mosquitoes^28^. Critically, a higher mortality rate in competent vectors has obvious implications for epidemiology. Consequently, while the mosquitoes in the 25 °C treatment were competent to transmit JEV, the impact on longevity due to infection may limit the ability of this virus to emerge in temperate areas. Specifically, as the virus may not be able to maintain consistent transmission cycles as it may negatively impact the persistence of a vector reservoir. At 20 °C no gross cellular damage was recorded during histological analysis. However, viral infection of mosquitoes has been shown to impact cellular and organ integrity, which may impact mortality rates and indeed vector competence, should salivary glands be damaged^51,52^. As we have recorded higher mortality rates in both of our treatment groups than would be expected, and that no cellular damage was recorded in the 20 °C group, it is unlikely that cellular damage is contributing to the increased mortality rate.

Understanding the susceptibility of naïve vector communities to emerging infection facilitates the design of robust, prophylactic disease intervention strategies. UK mosquitoes have been shown to be competent vectors for a number of arboviruses not currently present in the UK. Our results contribute to this expanding dataset and indicate that after fourteen days JEV genotype III can infect the temperate UK mosquito *Cx. pipiens*. However, environmental conditions required for competence, increase virulence for the vector, reflected by increased mortality, and may therefore influence JEV transmission, should the virus emerge in the UK.

## Supplementary Information

Supplementary Information.

## Data Availability

Data has been made available on FigShare under the title “The capacity of the temperate UK mosquito *Culex pipiens pipiens* to act as a vector for Japanese encephalitis virus” https://doi.org/10.6084/m9.figshare.13326683.
