# Supplementary material for: Temperate conditions restrict Japanese encephalitis virus infection to the mid-gut and prevents systemic dissemination in Culex pipiens mosquitoes
Source: Sci Rep. 2021 Mar 17;11:6133. doi: 10.1038/s41598-021-85411-2 (PMC7971067; doi:10.1038/s41598-021-85411-2)

**Title: Temperate conditions restrict Japanese encephalitis virus infection to the mid-gut and prevents systemic dissemination in *Culex pipiens* mosquitoes**

**Authors:** Arran J. Folly *^(1)^, Daniel Dorey-Robinson ^(1,2)^, Luis M. Hernandez-Triana ^(1)^, Stuart Ackroyd ^(3)^, Beatriz Vidana ^(3 4)^, Fabian Lean ^(3)^, Daniel Hicks ^(3)^, Alejandro Nuñez ^(3)^, Nicholas Johnson ^(1,5)^

**Affiliations**:

(1) Arbovirus Research Team, Virology Department, Animal and Plant Health Agency, Woodham Lane, Addlestone, Surrey, KT15 3NB, United Kingdom

(2) Current address: Pirbright Institute, Ash Road, Woking, Surrey, GU24 ONF, UK

(3) Pathology Department, Animal and Plant Health Agency, Addlestone, Surrey, KT15 3NB,

(4) Current Address: Bristol Veterinary School, University of Bristol, Langford House, Langford, Bristol, BS40, 5DU, UK

(5) Faculty of Health and Medicine, University of Surrey, Guildford, Surrey, GU2 7XH, UK

***Corresponding author**: Arran J. Folly [arran.folly@apha.gov.uk](mailto:arran.folly@apha.gov.uk)

**Supplementary data**

**Table S1.** Average climate data for the England (South East and Central South) from 1981 to 2010. Data obtained from Met Office statistics at <https://www.metoffice.gov.uk/research/climate/maps-and-data/uk-climate-averages/gcpevmgzn> (Accessed 08/06/2020).

| **Month** | **Maximum temperature (°C)** | **Minimum temperature (°C)** | **Days of air frost (days)** | **Sunshine (hours)** | **Rainfall (mm)** | **Days of rainfall ≥1 mm (days)** |
| --- | --- | --- | --- | --- | --- | --- |
| January | 7.5 | 1.7 | 10.1 | 58.8 | 80.1 | 12.6 |
| February | 7.7 | 1.4 | 10.4 | 78.8 | 55.1 | 9.7 |
| March | 10.5 | 3 | 6.2 | 114.3 | 57.6 | 10.6 |
| April | 13.2 | 4.3 | 3.4 | 169.8 | 53.7 | 9.7 |
| May | 16.7 | 7.3 | 0.6 | 200.7 | 54.6 | 9.3 |
| June | 19.6 | 10.1 | 0 | 201.4 | 51.2 | 8.4 |
| July | 22 | 12.2 | 0 | 214.9 | 51.7 | 8.1 |
| August | 21.8 | 12.1 | 0 | 203.9 | 57.4 | 8.3 |
| September | 18.9 | 10.1 | 0.1 | 149.1 | 62.3 | 9 |
| October | 14.8 | 7.4 | 1.4 | 113.4 | 92.8 | 11.9 |
| November | 10.7 | 4.2 | 5.1 | 71.4 | 87.3 | 12 |
| December | 7.9 | 2 | 9.9 | 51.1 | 83.8 | 12 |
| Annual | 14.3 | 6.3 | 47.1 | 1627.7 | 787.6 | 121.6 |

**Figure S1.**

Examples of Japanese encephalitis virus isolation in Vero cells from infected mosquito body or leg homogenates and saliva expectorates. Cells were fixed in 80% acetone and stained with crystal violet


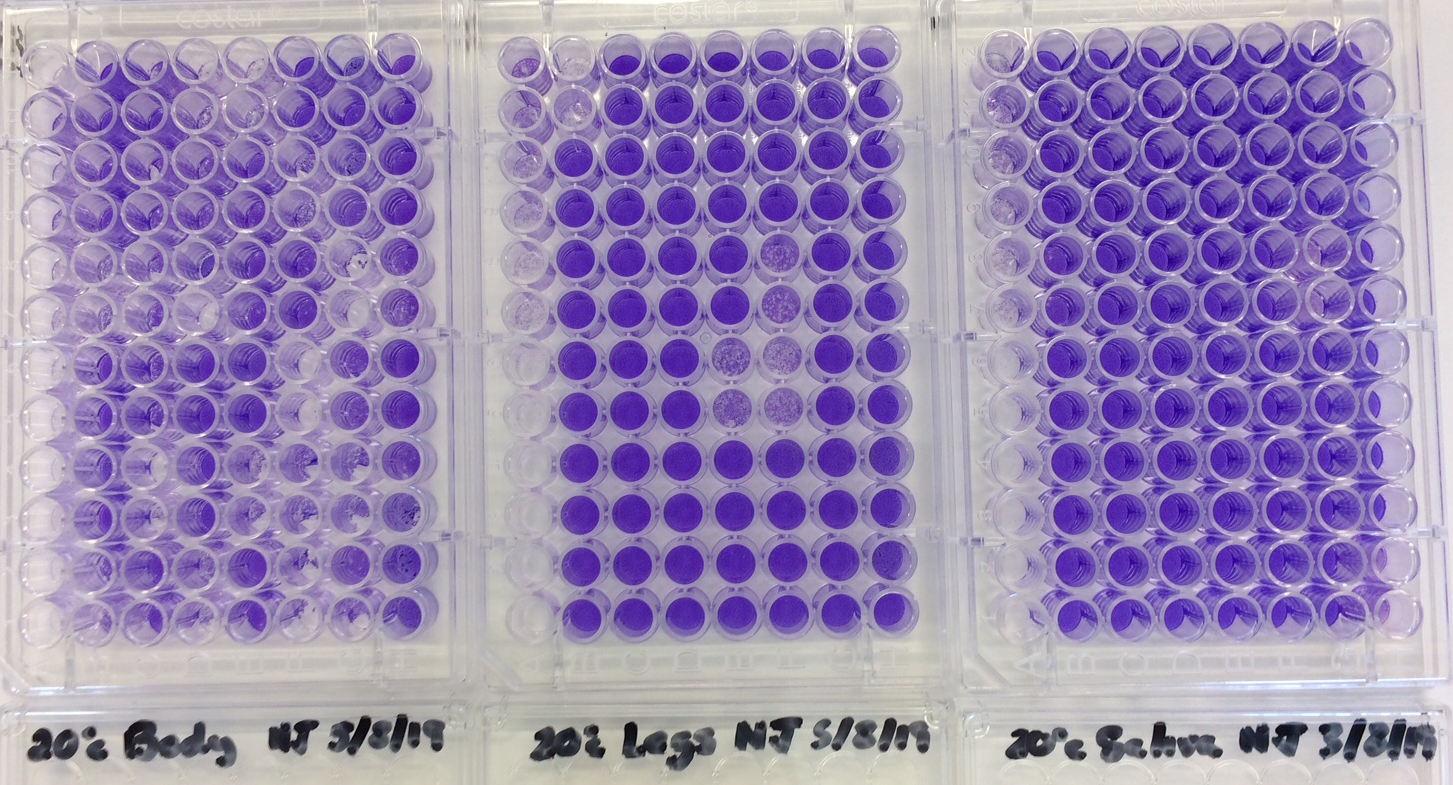

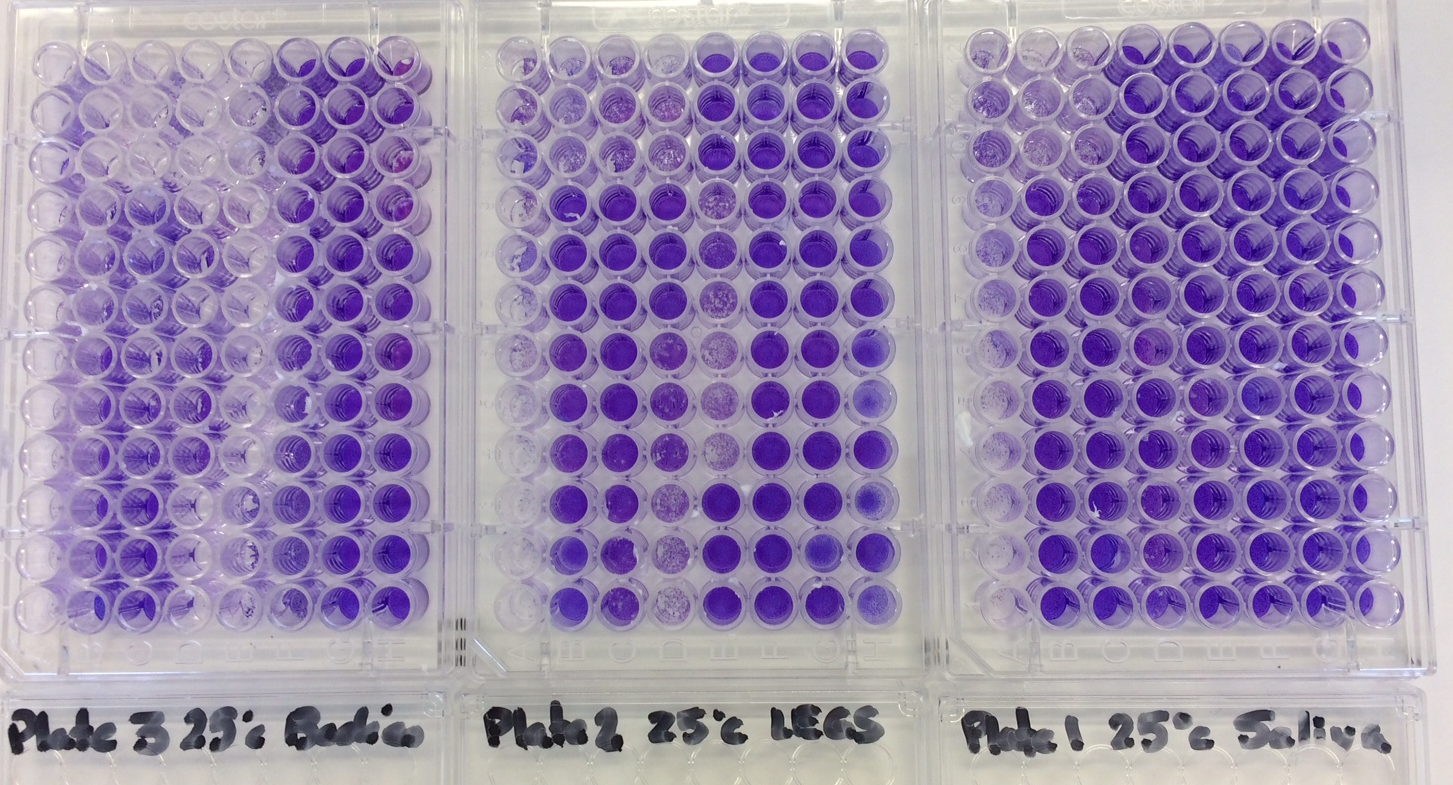


**Figure S2**. Read coverage depth for two JEV SA-14 (GenBank accession number KU323483, length = 10,977 bp, GC content = 51.43%) consensus sequences generated from two expectorated *Cx. pipiens* saliva samples 14 days post inoculation. The red line indicates average read coverage depth.

**
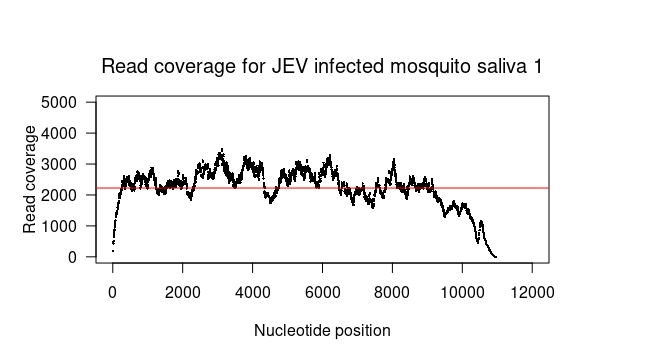

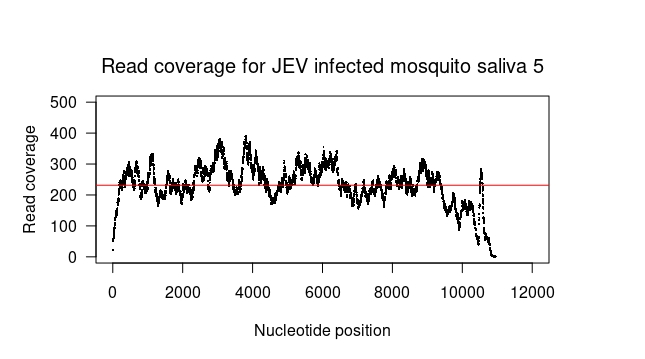
**

**Figure S3. Isotype control immunohistochemical labelling of experimental JEV infection*.*** Serial section of mosquitoes as per figure 4. Images taken at 20x maginifcation.


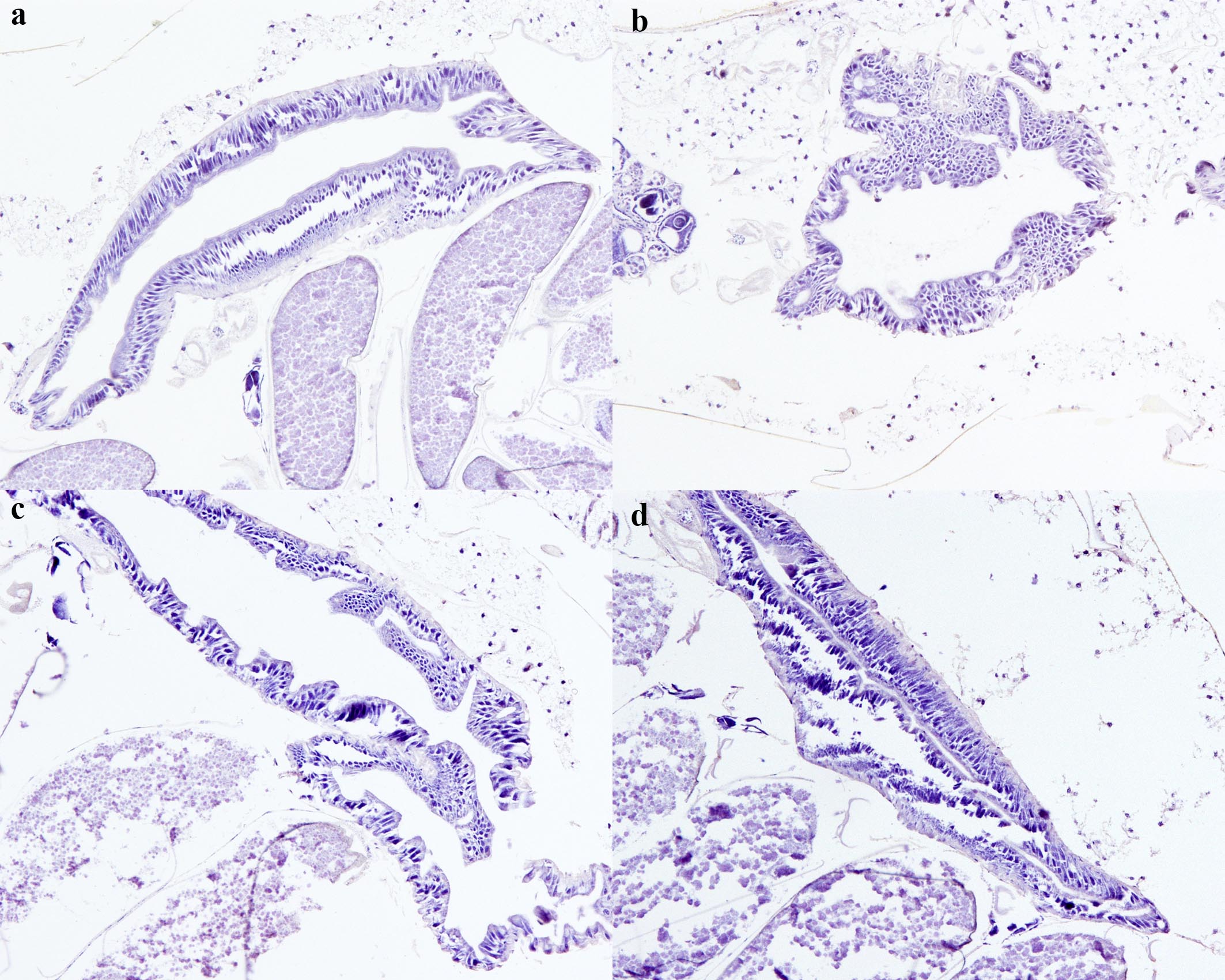

Supplement: Supplementary file 1 — Supplementary Information. [file 41598_2021_85411_MOESM1_ESM.docx]
